# Prelinearized Class-B Power Amplifier for Piezoelectric Transducers and Portable Ultrasound Systems

**DOI:** 10.3390/s19020287

**Published:** 2019-01-12

**Authors:** Hojong Choi

**Affiliations:** Department of Medical IT Convergence Engineering, Kumoh National Institute of Technology, 350-27 Gumi-daero, Gumi 39253, Korea; hojongch@kumoh.ac.kr; Tel.: +82-054-478-7782

**Keywords:** class-B power amplifier, prelinearizer, portable ultrasound systems

## Abstract

Portable ultrasound systems typically suffer from unwanted heat and limited battery life, resulting in reduced system performance or the applicable number of piezoelectric transducer elements. This can be a bottleneck in widely used portable ultrasound systems. Class-A power amplifiers are typically used in portable ultrasound systems. However, unwanted heat dissipation needs to be reduced by using large cooling fans and heat pipe structures. To reduce unwanted heat, class-B power amplifiers may be a possible solution. However, the non-linearity of class-B power amplifiers could limit their integration with piezoelectric transducers because non-linearity in the high-voltage output of the power amplifiers deteriorates the sensitivity of portable ultrasound systems. To improve the linearity of the power amplifier, we developed prelinearized class-B power amplifiers for piezoelectric transducers and portable ultrasound systems. To verify our proposed method, we compared the performances of class-B and prelinearized class-B power amplifiers in their pulse-echo responses. Therefore, prelinearized class-B power amplifiers are a possible solution to produce better echo signal performance in piezoelectric transducers and portable ultrasound systems.

## 1. Introduction

Portable ultrasound systems have been used for non-destructive and diagnostic applications in structural health monitoring, ambulances, and emergency rooms [1,2]. However, these systems can suffer from heat and battery issues [1]. The most significant heat and battery issues are initiated by the analog transmitters of the portable ultrasound systems [3,4]. In analog transmitters, power amplifiers are one of the most influential sources that generate unwanted heat [5]. Commercial ultrasound companies have used large cooling fans and heatsink systems to reduce the heat in benchtop ultrasound machines [6].

In commercial ultrasound benchtop products, additional cooling systems are integrated with ultrasound systems to avoid performance degradation, thus increasing system reliability [4]. However, it is challenging to use these solutions in portable ultrasound systems owing to their limited size and the structure of the systems and piezoelectric transducers.

Ultrasound companies are striving to resolve heat problems in portable ultrasound systems. In analog transmitters, Class-A power amplifiers, which are linear power amplifiers, are used conducting all the time in the transient response, generating heat in ultrasound systems [7]. Additionally, large cooling fans can generate undesired mechanical motor noise that affects the performance of piezoelectric transducers in motor-controlled ultrasound systems. When high-voltage or high-power energy produced by the power amplifiers is delivered to the piezoelectric transducer load, the heat usually affects the resistance, capacitance, and inductance values of the power transistors, resistors, capacitors, and inductors.

Instead of class-A power amplifiers, non-linear power amplifiers were recently studied for piezoelectric transducer applications [8,9,10]. For a 100-kHz piezoelectric transducer, a class-D amplifier using power metal-oxide-semiconductor field-effect transistor (MOSFET) bridge components was proposed to increase the power for the capacitive load [11]. For magnetic resonance imaging-guided high-intensity focused ultrasound, the class-DE driver was proposed for a 1-MHz piezoelectric transducer [8]. A class-E inverter was also proposed for a Langevin piezoelectric transducer to find the optimal driving frequency of the inverter [9]. The non-linear power amplifiers have advantages in reducing direct current (DC) consumption, which can lower unwanted heat and increase battery life [12]. However, nonlinearity in the output of the power amplifiers causes image-resolution degradation or increases signal distortion [6]. In particular, harmonic imaging methods or modulated excitation methods are very sensitive to signal distortions because of the wide bandwidth of the piezoelectric transducers [13,14]. The transmitted signals with signal distortion components need to be suppressed before receiving the echo signals detected by the piezoelectric transducers [15]. Push-pull type class-B power amplifier utilizes the transformer components for the input and output ports because of the positive and negative differential signals [16]. Therefore, the push-pull type class-B power amplifier is undesirable for the piezoelectric transducer which is sensitive to long cables. Additionally, the echo signal could be merged with the discharged pulses for the piezoelectric transducer which has fast pulse-echo responses. Therefore, single-ended power amplifier is also useful for such a kind of piezoelectric transducer.

**Figure 1:**
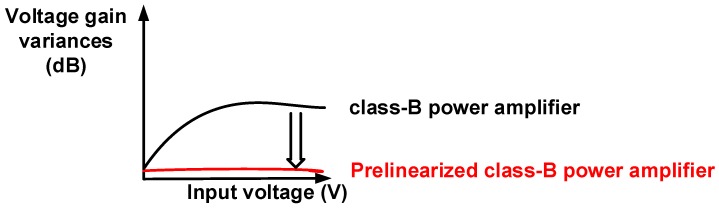
Concept of voltage gain variances vs. input voltage graph of class-B power amplifier and prelinearized class-B power amplifier.

The class-A and class-B power amplifiers can be categorized depending on the DC operating points rather than the architectures [7,17]. Compared to a class-A power amplifier, the class-B power amplifier has relatively higher signal distortions and lower DC current consumption because the DC operating point of the class-B power amplifier is lower than that of the class-A power amplifier [17,18]. To improve the linearity of the power amplifier, a negative feedback loop network can be utilized. However, this scheme can critically reduce the voltage gain of the class-B power amplifier [19]. This is undesirable for power amplifiers used in piezoelectric transducers, which typically require high-power transduction. To reduce the non-linearity of the class-B power amplifier, we developed a prelinearized class-B power amplifier, as depicted in Figure 1. The linearity characteristics of the power amplifiers are improved by reducing the voltage gain variances [20]. Therefore, a stable voltage gain of the power amplifier over the input voltage range can be achieved.

A high-voltage transistor simulation library is still one of the important research and manufacturing issues for high-voltage applications such as medical imaging modalities and automobile applications because it has been developed to provide more precise simulation data [12,21]. However, a library for the high-voltage MOSFET is unable to provide accurate equivalent circuit design models for power amplifier design, which requires subdecibel accuracy [16]. Therefore, power amplifier designs are typically implemented on printed circuit boards in order to obtain their measured performance [16,17]. Section 2 describes the design architecture and operating mechanisms of class-B and prelinearized class-B power amplifiers. Section 3 provides the measurement results and discussion of the class-B and prelinearized class-B power amplifiers for linearity comparisons. Section 4 presents the conclusion of the paper.

## 2. Materials and Methods

**Figure 2:**
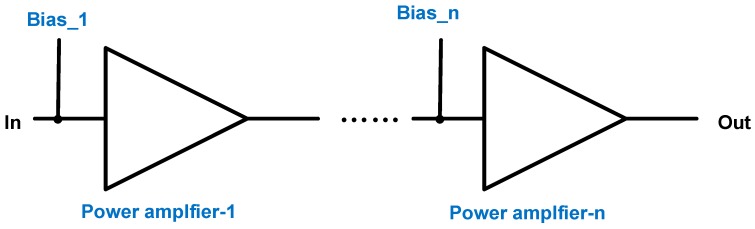
Schematic diagrams of multistage class-B power amplifier with bias points.

Figure 2 shows multistage power amplifiers with bias circuits (Bias_1 and Bias_n). The DC bias voltages in the bias circuits are important to provide steady DC operating points, especially for class-B power amplifiers. This is because the transistor devices are the main devices of the power amplifiers, which are operated in distortion regions [7]. This affects the performances of the power gain, bandwidth, and signal distortion [22]. Compared to the class-A power amplifier, the class-B power amplifier needs to use a higher input voltage to obtain the same output voltage for the piezoelectric transducers since the output voltage amplitude is related to the sensitivity of the piezoelectric transducer [22].

**Figure 3:**
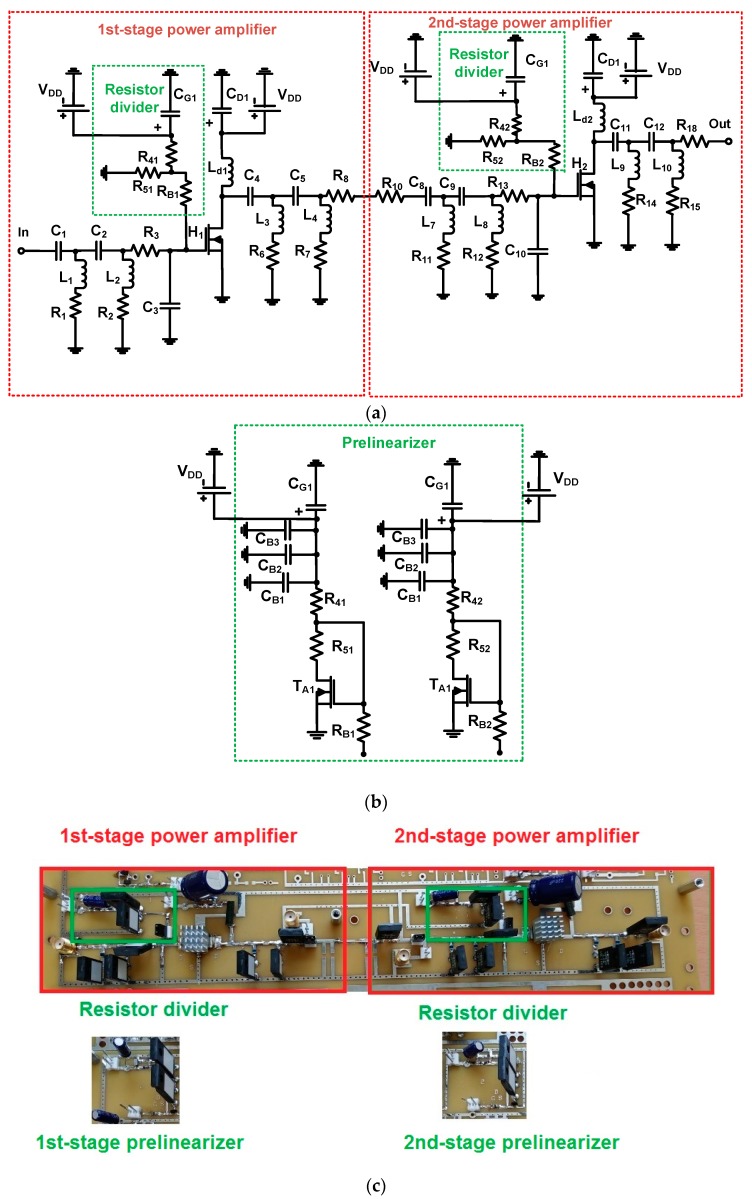
Schematic diagrams of (**a**) class-B power amplifier, (**b**) prelinearized class-B power amplifier, and (**c**) fabricated printed circuit boards.

Figure 3 shows schematic diagrams of the class-B and prelinearized class-B power amplifiers on a fabricated printed circuit board. For the class-B power amplifier, the biasing circuits could be more sensitive for the amplifier performance because the class-B power amplifier operates in lower DC bias regions compared to the class-A power amplifier [7]. Therefore, a prelinearizer circuit needs to be considered for factors such as the DC current consumption or voltage gain.

For our power amplifier design, all electronic components, which are high-voltage tolerant, were implemented on a printed circuit board. To minimize the parasitic impedance, surface-mounted capacitors and inductors (except for the power film resistors and electrolytic capacitors) were used.

As shown in Figure 3a, a two-stage power amplifier with a typical resistor divider was designed for a class-B power amplifier. In the two-stage power amplifier, each resistor divider was composed of two resistors (R_4_ and R_5_) and a blocking AC resistor (R_B1_), and these fed into the power transistors (H_1_ and H_2_), respectively. Bias gate voltages were applied to each stage of the power amplifier. The main transistor of the power amplifier was a high-voltage MOSFET device (H_1_ and H_2_, PD57018-E, STMicroelectronics, Inc., Geneva, Switzerland). All components of the power amplifiers were high-voltage tolerant.

As shown in Figure 3b, a prelinearizer circuit was implemented to replace the resistor divider in order to improve the linearity of the two-stage power amplifiers. The large input voltage signals up to 5V_p-p_ could actually pass through the power film resistors (R_B1_ and R_B2_) such that they affected the stability of the DC power supply. An additional three capacitors (C_B1_, C_B2_, and C_B3_) with one power film resistor (R_B1_) were used as a low-pass filter. Figure 3c shows the implemented two-stage class-B power amplifiers with resistor divider and prelinearizer circuits on the printed circuit board. To reduce the thermal heat, the aluminum heat-sinks were attached to the top side even though they could not completely remove the heat.

**Figure 4:**
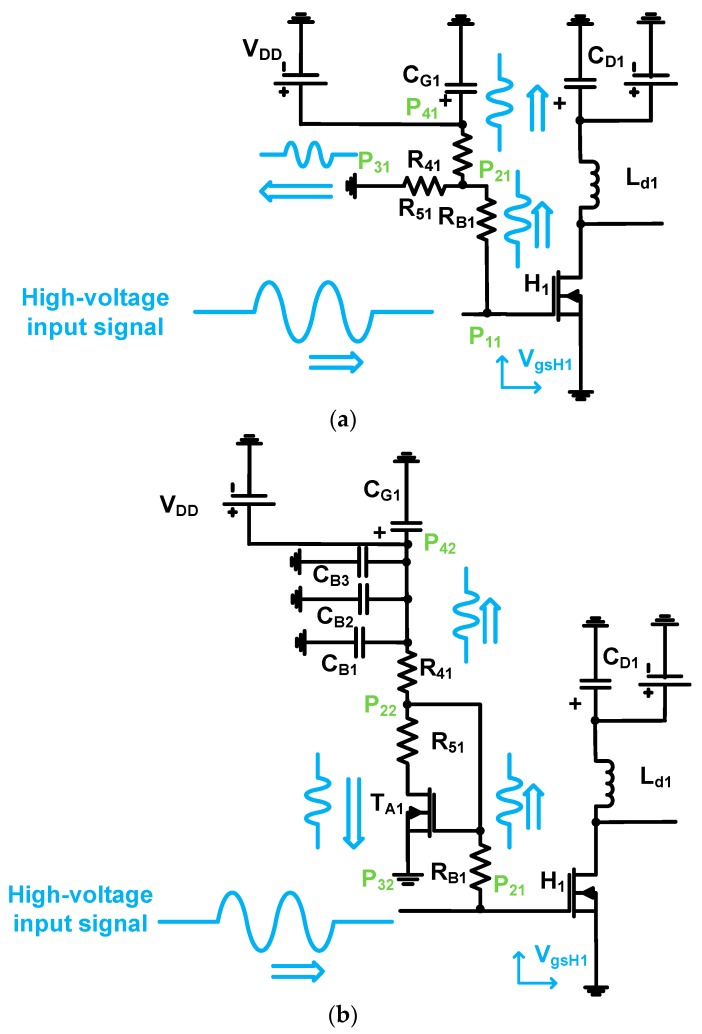
Working mechanisms of (**a**) class-B power amplifier and (**b**) prelinearized class-B power amplifier.

In Figure 4, the working mechanisms of the class-B power amplifier with a resistor divider and class-B power amplifier with prelinearizer are described to elucidate a high-voltage input signal flow and analyze the predicted behavior of the circuits. As described above, the class-B power amplifier needs to use high-voltage and high-frequency input signals to obtain adequate output voltage signals for the piezoelectric transducers because the bias voltage point of the class-B power amplifier is lower than that of the class-A power amplifier, resulting in lower output voltages [7].

As shown in Figure 4a, a high-voltage and high-frequency input signal cannot be blocked by the resistor (R_B1_). Thus, the input signals coming from the input pass through the bias resistor (R_B1_) and sink the current to the resistor (R_41_) in the resistor divider. High temperature caused by the high-voltage and high-frequency input signals can also make the resistance values of the resistor divider unstable. Therefore, the gate-source voltage (V_gsH1_) of the power transistor (H_1_) can be affected such that the output voltage of the power amplifier can be changed accordingly.

As shown in Figure 4b, the high-voltage and high-frequency input signal also passes through the bias resistor (R_B1_). Then, this input signal passes two different paths of the transistor (T_A1_, P_22_ to P_42_) and three shunt capacitors (C_B1_, C_B2_, and C_B3_, P_22_ and P_32_). These shunt capacitors suppress the unwanted high-voltage and high-frequency input signals to stabilize the DC voltage (V_DD_) of the power supply. Compared to the class-B power amplifier, there are more current sinks from the high-voltage transistor (T_A1_) such that the gate-source voltage (V_gsH1_) drop of the power transistor (H_1_) can be changed accordingly.

**Figure 5:**
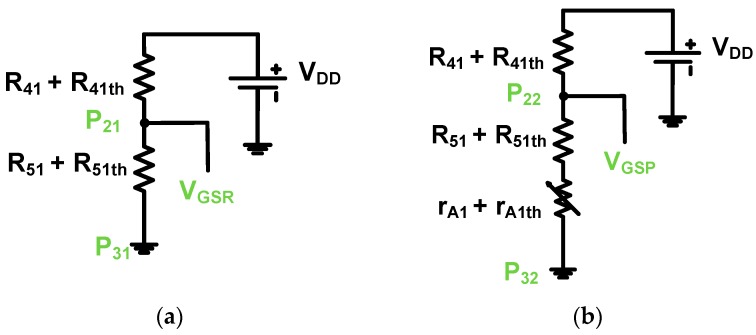
Equivalent circuit models of (**a**) resistor divider and (**b**) prelinearizer of class-B power amplifier.

As the input voltage increases, the high-voltage transistor (T_A1_) in the prelinearizer works as a variable resistor through the power supply (V_DD_). The voltage drop through the transistor (P_22_ to P_32_) depends on how much voltage is applied to the drain-source resistance. The gate-source voltage drop of the high-voltage transistor (V_gsH1_) can also be considered by the bias voltages owing to temperature variations. Thus, we predict that a more linearized voltage output signal of the prelinearized class-B power amplifier can be obtained owing to the increased bias voltage (V_gsH1_). However, we expect that the current consumption of the prelinearized class-B power amplifier may be higher than that of the class-B power amplifier. Figure 5 shows equivalent circuit models of the resistor divider and prelinearizer for the bias voltage analysis.

If we consider that thermal resistances of the power film resistors and drain-source resistance of the high-voltage transistor can affect the bias voltages of each power amplifier, the gate-source bias voltages (V_GSR_ and V_GSP_) of the class-B power amplifier and prelinearized class-B power amplifier can be expressed as (1) and (2).

(1) $$V_{\mathrm{GSR}}=\frac{R_{41}+R_{41\mathrm{th}}}{R_{41}+R_{41\mathrm{th}}+R_{51}+R_{51\mathrm{th}}}V_{\mathrm{DD}}$$

(2) $$V_{\mathrm{GSP}}=\frac{R_{41}+R_{41\mathrm{th}}+r_{A1}+r_{A1\mathrm{th}}}{R_{41}+R_{41\mathrm{th}}+R_{51}+R_{51\mathrm{th}}++r_{A1}+r_{A1\mathrm{th}}}V_{\mathrm{DD}}$$

where r_A1_ is the drain-source resistance of the high-voltage transistor (T_A1_); R_41th_, R_51th_, and r_A1th_ are the thermal resistances of R_41_, R_51_, and r_A1_; and V_DD_ is the supply voltage of the power amplifiers.

As shown in (2), the gate-source voltage of the prelinearized class-B power amplifier is affected by the high-voltage transistor’s drain-source resistance (r_A1_). In the next chapter, we will measure and compare the performances to verify the linearity characteristics of the class-B power amplifier and prelinearized class-B power amplifier.

## 3. Results

### 3.1. Performance Comparison of Class-B Power Amplifier and Prelinearized Class-B Power Amplifier

**Figure 6:**
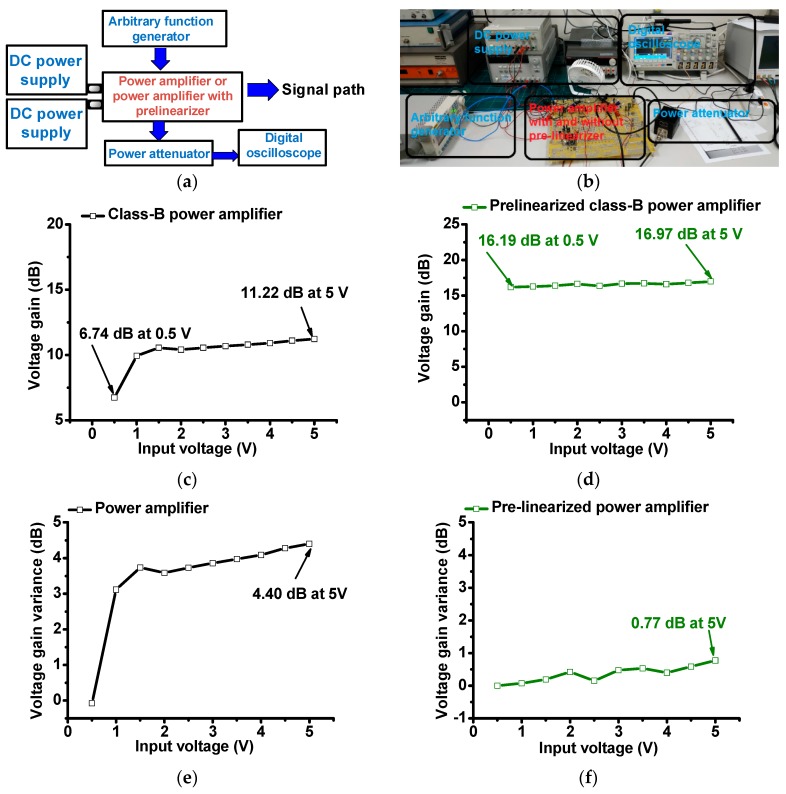
(**a**) Schematic diagram and (**b**) photo of measurement setup; voltage gain vs. input voltage of (**c**) power amplifier and (**d**) prelinearized power amplifier; voltage gain deviation vs. input voltage of (**e**) power amplifier and (**f**) prelinearized power amplifier.

In order to verify our proposed idea, we checked and compared the performances of the class-B power amplifier and prelinearized class-B power amplifier. Figure 6 shows the measurement setup of the class-B power amplifier and prelinearized class-B power amplifier for voltage gain, voltage gain variances, and DC current consumption. For every electronic component, there is a thermal impedance. The thermal impedances are typically proportional to the temperature across the electronic components [12]. This phenomenon can affect the piezoelectric transducer performance of portable ultrasound systems.

In Figure 6a, a sine pulse generated by the arbitrary function generator (DG5071, Rigol Technologies, Beijing, China) is amplified by the designed power amplifiers. The power amplifiers were biased by the first DC power supply and second DC power supply. The amplified signals were attenuated by a 50-W power attenuator to measure the voltage gain in the digital oscilloscope (MSOX4154A, Keysight Technology, Santa Clara, CA, USA).

In Figure 6b,c, the measured voltage gain vs. input voltage graphs of the class-B and prelinearized class-B power amplifiers were plotted to check the linearity characteristics. The maximum output voltage gains of the class-B power amplifier and prelinearized amplifier are 11.22 dB and 16.97 dB at a 5-V_p-p_ input voltage, respectively. After the 1.5-V_p-p_ input voltage, the voltage gain of the class-B power amplifier was stabilized. Figure 6d,e show the normalized voltage gain variances vs. input voltages of the class-B power amplifier and prelinearized class-B power amplifier in order to check the linearity characteristics.

As shown in Figure 6d,e, the voltage gain variance of the prelinearized class-B power amplifier (0.77 dB) is less than that of the class-B power amplifier (4.40 dB) at a 5-V_p-p_ input. Additionally, the gain variance of the prelinearized class-B power amplifier is flatter than that of the class-B power amplifier over the input voltage ranges. These measurement data are meaningful because the prelinearizer can increase the linearity of the class-B power amplifier, thus reducing the output voltage signal distortions of the power amplifier.

**Figure 7:**
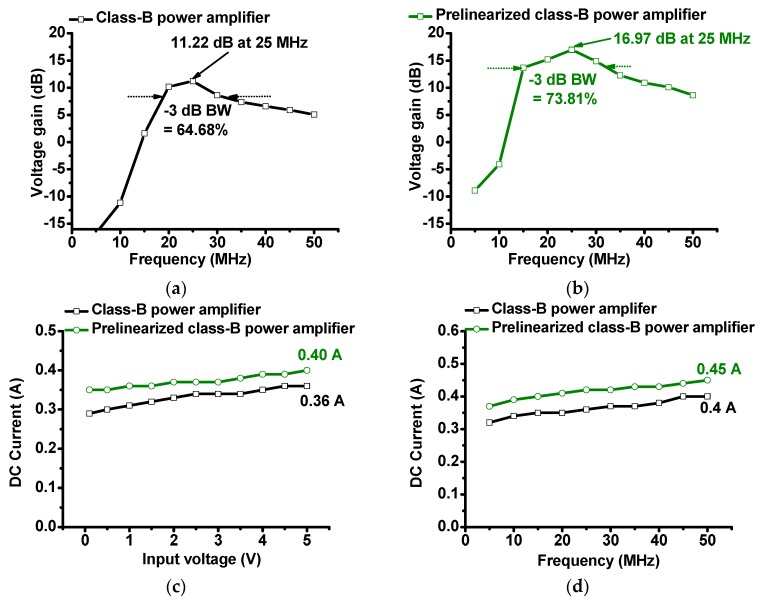
Voltage gain vs. frequency of (**a**) class-B power amplifier and (**b**) prelinearized class-B power amplifier. (**c**) Direct current (DC) consumption vs. input voltage of class-B power amplifier and class-B prelinearized power amplifier. (**d**) DC current consumption vs. input frequency of class-B power amplifier and class-B prelinearized power amplifier.

As shown in Figure 7a,b, the measured voltage gain of the prelinearized class-B power amplifier (16.97 dB) is higher than that of the class-B power amplifier (11.22 dB), and it is also higher than that of the power amplifier over the frequency ranges. The −3-dB bandwidth of the prelinearized class-B power amplifier (73.81%) is also slightly wider than that of the class-B power amplifier (64.68%). Compared to the class-A power amplifier, the class-B power amplifier is useful in reducing the DC current consumption. We measured the DC current consumption vs. input voltages and frequencies of the class-B power amplifier and prelinearized class-B power amplifier, respectively, in Figure 7c,d.

The DC current consumption of the prelinearized class-B power amplifier is around 10% higher (0.45 A) than that of the class-B power amplifier (0.4 A) at 50 MHz. Therefore, we conclude that the prelinearized class-B power amplifier produces lower gain deviations, higher voltage gains, and higher DC current consumption.

### 3.2. Performance Comparison of Pulse-Echo Responses

The pulse-echo responses are a typical performance measurement for developed piezoelectric transducers and ultrasound electronic components [23,24]. In typical ultrasound systems, a class-A power amplifier, which is a linear power amplifier, has been used [4]. The pulse-echo responses were measured because class-B power amplifiers are non-linear power amplifiers such that the proper pulse-echo responses need to be checked before integrating the device with other ultrasound system components. Therefore, the designed class-B and prelinearized class-B power amplifiers need to be confirmed by examining the piezoelectric transducer performances.

**Figure 8:**
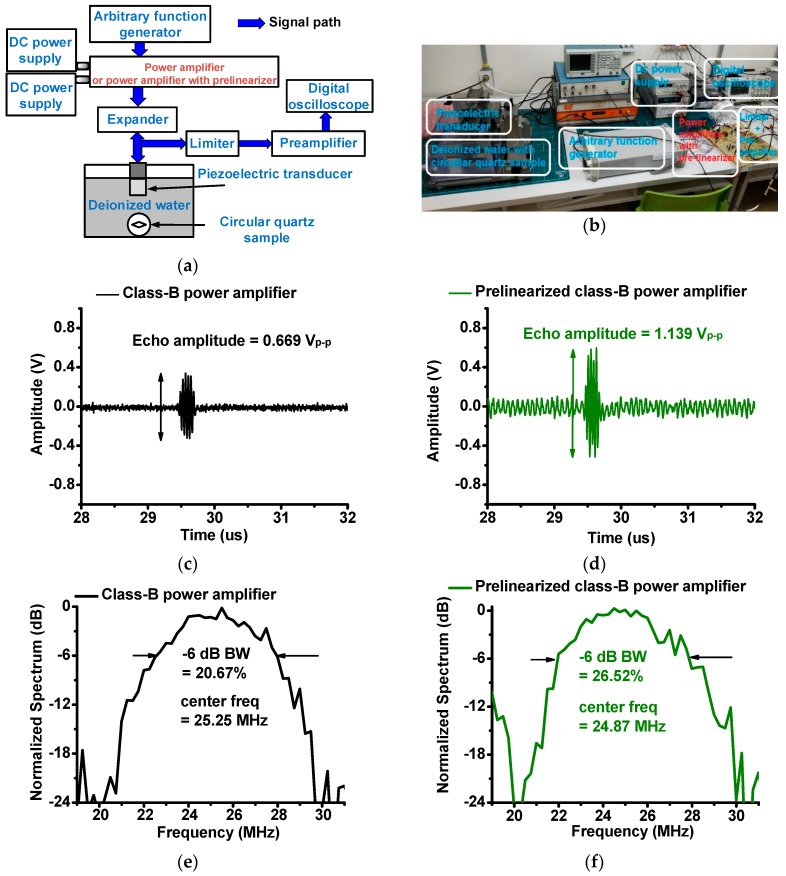
(**a**) Measurement setup and (**b**) photo of pulse-echo response. Echo signal amplitude of piezoelectric transducer when using (**c**) class-B power amplifier and (**d**) prelinearized class-B power amplifier. Normalized spectrum of piezoelectric transducer when using (**e**) class-B power amplifier and (**f**) prelinearized class-B power amplifier.

Figure 8a shows the measurement setup of the pulse-echo responses. The sine pulses generated from the arbitrary function generator (DG5071) were fed into the designed power amplifiers, which were biased by two DC power supplies. The amplified sine pulses triggered a focused piezoelectric transducer through an expander that is composed of a single cross-coupled switching diode pair.

The generated ultrasonic pulses were sent and reflected by a circular quartz sample in deionized water. The received echo signals from the piezoelectric transducer were then sent through a limiter, which is composed of a 50-Ω resistor shunt with a cross-coupled switching diode pair, amplified by a preamplifier (AU-1354, L3 Narda-MITEQ, Inc., Hauppauge, NY, USA). The transient echo signals were displayed on a 1-GHz digital oscilloscope (MSOX4154A) to plot the echo amplitudes and spectrum data of the acoustic echo signals.

The echo signal amplitude and its spectrum data are related to the sensitivity and resolution of the piezoelectric transducer [25]. A comparison of the echo signal amplitude data when using the class-B and prelinearized class-B power amplifiers is shown in Figure 8. The size of each piezoelectric array transducer is limited such that maximum applied voltages are also influenced by the sizes of the piezoelectric array transducers [26,27]. The sensitivity of the piezoelectric array transducers could be low, accordingly. For the piezoelectric transducers with low sensitivity, the echo signal usually cannot be detectable when there are high noise signals which might be generated from the mechanical motors or transmitter electronics in the ultrasound machines [26,28]. Since the prelinearized class-B power amplifier produced higher voltage gain compared to the class-B power amplifier, we compared the echo signal amplitudes of the pizeoelectric transducers when using the class-B and prelinearized class-B power amplifiers, as shown in Figure 8c,d. The peak-to-peak amplitude of the echo signals generated by the piezoelectric transducers when using the prelinearized class-B power amplifier (1.139V_p-p_) was higher than that when using the class-B power amplifier (0.669V_p-p_). As shown in Figure 8d, the echo signal amplitude of the piezoelectric transducer when using the prelinearized class-B amplifier was improved compared to that when using the class-B amplifier alone. The noise signal fluctuation was also increased such that the signal-to-nose ratio of the echo signals could have no improvement. However, prelinearized class-B power amplifier would be more useful to increase the echo amplitudes of the piezoelectric transducer with low sensitivity for the portable ultrasound machines.

Figure 8e,f, show the center frequencies and −6-dB bandwidths of the echo signals of the piezoelectric transducer when using the class-B power amplifier and prelinearized class-B power amplifier. The −6-dB bandwidth of the echo signal when using prelinearized a class-B power amplifier (26.52%) is slightly improved compared to that of the class-B power amplifier (20.67%). The center frequencies of the power amplifiers are similar (25.25 MHz for the class-B power amplifier and 24.87 MHz for the linearized class-B power amplifier). Therefore, we conclude that the improved output voltage amplitudes of the prelinearized class-B power amplifier can increase the sensitivity of the piezoelectric transducer for the portable ultrasound machines.

## 4. Conclusions

Portable ultrasound machines suffer from unwanted heat generated from class-A power amplifiers, resulting in decreased piezoelectric transducer performance during long-term operations. To reduce unwanted heat, heat sinks with large cooling fan systems a normally inserted in bench-top ultrasound machines. However, portable ultrasound machines have a limited structural size. The class-A power amplifier, which generates high heat, is free from non-linearity. For this reason, we proposed a prelinearized class-B power amplifier to improve the linearity performance. To verify the capability of the prelinearized class-B power amplifier, we tested the voltage gain and voltage gain variances vs. input voltage and measured the DC current consumption vs. input voltage of a class-B power amplifier and a prelinearized class-B power amplifier.

The voltage gains of the class-B power amplifier and prelinearized power amplifier were 11.2 dB and 16.97 dB at a 5-V_p-p_ input voltage. The voltage gain variances of the class-B power amplifier and prelinearized power amplifier were 4.40 dB and 0.77 dB at a 5-V_p-p_ input voltage. The DC current consumptions of the class-B power amplifier and prelinearized power amplifier were 0.36 A and 0.40 A at a 5-V_p-p_ input voltage. Therefore, the prelinearized class-B power amplifier improved the linearity while increasing the DC current consumption.

Finally, we tested the pulse-echo responses in an ultrasound system. We obtained a higher echo signal amplitude when using the prelinearized class-B power amplifier (1.139V_p-p_) compared to a class-B power amplifier (0.669V_p-p_). Therefore, we conclude that the prelinearized class-B power amplifier is a solution for portable ultrasound machines.
